# Deciphering the Glycoproteomic Landscape of Mood Disorders: Unveiling Molecular Association Between CDG and Depression Resilience

**DOI:** 10.21203/rs.3.rs-6882753/v1

**Published:** 2025-07-10

**Authors:** Tamas Kozicz, Rohit Budhraja, Graeme Preston, Neha Joshi, Irena Muffels, Gustavo Turecki, Mark Frye, Marin Veldic, Eva Morava, Akhilesh Pandey

**Affiliations:** Icahn School of Medicine at Mount Sinai; Mayo Clinic; Icahn School of Medicine at Mount Sinai; Mayo Clinic; Icahn School of Medicine at Mount Sinai; McGill University; Mayo Clinic; Mayo Clinic; Icahn School of Medicine at Mount Sinai; Mayo Clinic

**Keywords:** Congenital disorders of glycosylation, bipolar disorder, major depression, manic mood state, depressed mood state, protein N-glycosylation, LC-MS/MS, glycoproteomics, postmortem brain, dorsolateral prefrontal cortex

## Abstract

The lack of a molecular understanding of mood disorders has impeded progress in diagnosis and treatment. Glycosylation may provide insights into the complex mechanisms underlying these conditions. We conducted N-glycoproteomic analysis on dorsolateral prefrontal cortex samples from individuals with major depressive disorder (MDD) and bipolar disorder (BD), in depressive or manic states at death. Additionally, we examined depression prevalence in congenital disorders of glycosylation (CDG) through a literature review and assessment of 110 CDG patients. Glycoproteomic analysis revealed a significant increase in protein glycosylation in individuals with MDD relative to both controls and individuals with BD. Depression prevalence was lower in our pediatric and adult cohort of individuals with CDG. These results suggest brain glycosylation changes may play a role in mood disorder pathology and highlight the distinct biology of unipolar and bipolar depression. Our findings propose that impaired glycosylation may confer resilience to depression, offering potential therapeutic insights.

## Introduction

Mood disorders are a group of mental health illnesses characterized by fluctuations in a person’s emotional state (Aan het Rot, Hogenelst et al. 2012, Post, Altshuler et al. 2021). Depressive behavior is increasingly diagnosed, even in individuals of pediatric age, especially in chronic disease (Maltsev and Lorigan 2011). Among the most common mood disorders are major depressive disorder (MDD) (Kendler 2020, Post, Altshuler et al. 2021) and bipolar disorder (BD) (Miola, Fountoulakis et al. 2023, Nierenberg, Agustini et al. 2023). MDD presents with symptoms of persistent sadness, hopelessness, and reduced interest in daily activities, along with sleep disruption, appetite changes, concentration difficulties, fatigue, and sometimes thoughts of self-harm or suicide (Lewis and Piotrowski 1952, Liu, Blackwood et al. 2011). Conversely, BD is characterized by recurring swings in mood state, from to depressive to manic or hypomanic. The depressive phase – bipolar depression – often resembles symptoms of major (unipolar) depression (Nierenberg, Agustini et al. 2023). Manic episodes in bipolar disorder involve heightened energy, euphoria, impulsivity, reduced sleep, and may include psychosis and cognitive deficits (Hilty, Leamon et al. 2006).

The molecular complexities underlying mood disorders call for more personalized and targeted therapeutic approaches. Identifying specific molecular drivers of these disorders, discovering novel biomarkers (Cheng, Guan et al. 2020, Lee, Rhee et al. 2021, Jia, Hui et al. 2023), and developing precision therapies that address the underlying biology of these disorders remain significant challenges in the field of mental health medicine.

Protein N-glycosylation is a post-translational modification resulting in complex and highly regulated sugar chains ligated to asparagine residues on nascent proteins. These modifications are critical for both protein translation, maturation, and trafficking, as well as function. Proper protein N-glycosylation affects the nervous system in diverse ways, as glycoproteins are involved in regulating several neurological processes, including neural development, synaptic transmission, and ion transport (Scott and Panin 2014, Williams, Noel et al. 2022, Bradberry, Peters-Clarke et al. 2023). Differential glycosylation patterns have been observed in several neurological and neurodegenerative disorders, including schizophrenia (Abou-Abbass, Abou-El-Hassan et al. 2016, Zhang, Ma et al. 2020). Alterations in the N-glycosylation signatures could provide promising prospects for early diagnosis and post-treatment monitoring of patients with psychopathology (Stanta, Saldova et al. 2010).

Congenital disorders of glycosylation (CDG) are a large and diverse group of syndromes precipitating from genetic variants which impair protein N-glycosylation. Most individuals with CDG present with neurological symptoms and experience reduced physical strength, mobility, and satisfaction in social roles (Ligezka, Mohamed et al. 2022, Lam, Scaglia et al. 2024). These challenges raise important questions about the broader impact of CDG on mental health. Due to their chronic, complex disease pathologies, individuals with CDG have an increased risk of developing socio-emotional problems (van de Loo, van Dongen et al. 2013), however depression and depressive behavior in CDG patients are not well-documented.

To address this gap in research, we investigated the prevalence of depression in individuals with CDG by conducting a comprehensive literature review and analyzing patient records from 110 individuals with CDG. Following this, we performed a mass spectrometry-based N-glycoproteomics analysis on postmortem dorsolateral prefrontal cortex samples from 3 individuals with MDD, and 6 individuals with BD, 3 of whom were in a depressed mood state and 3 of whom were in a manic mood state at time of death. Our findings revealed a lower occurrence of depression among CDG patients and significant changes in glycosylation patterns in MDD and BDD, suggesting unique molecular disruptions that may contribute to their specific pathobiology.

## Results

### Comorbid depression in individuals with CDG

Our literature review identified publications describing 1,530 individuals with any one of 21 different CDG subtypes. Notably, only one individual described presented with comorbid depression, consistent with a depression prevalence of less than 0.007% in this population (Table 2). Next, we evaluated 110 patients with confirmed pathogenic CDG variants enrolled in the Frontiers of Congenital Disorders of Glycosylation Consortium (FCDGC) natural history study. This cohort included 110 individuals (18 adults and 92 children) aged 2 to 38 years, with any one of 30 CDG subtypes. Among them, 2 individuals presented with a confirmed diagnosis of comorbid depression (1.8% prevalence) (Table 3).

### Characterization of the DLPFC N-glycoproteome

**Figure 1:**
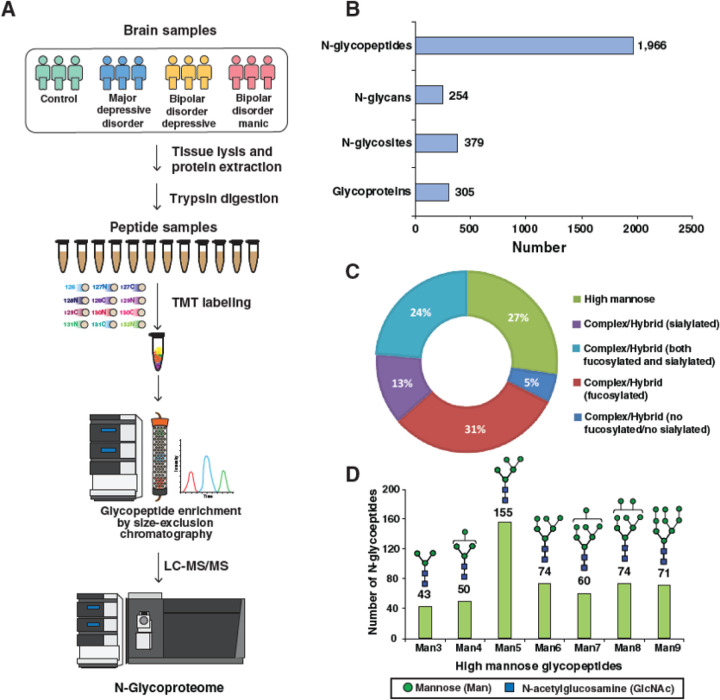
A schematic workflow for glycoproteomic analysis of postmortem brain samples from different mood disorders and global view of brain N-glycoproteome. **A.** The postmortem brain samples from individuals with major depressive disorder (MDD), bipolar depression who were in a depressed mood state (BDD), bipolar depression who were in a manic mood state (BDM), and controls were lysed, proteins were extracted and digested with trypsin, and equal amounts of peptides were labeled with tandem mass tags (TMT) prior to pooling. Pooled TMT labeled samples were used to enrich glycopeptides using size exclusion chromatography (SEC) for glycoproteomic analysis prior to liquid chromatography tandem mass spectrometry (LC-MS/MS). **B.** A bar graph representing the number of identified N-glycopeptides, N-glycan structures, N-glycosites, and N-glycoproteins in human brain samples. **C.** Pie chart representing the composition of the detected N-glycoproteome by glycan class. **D.** Bar chart displaying the number of glycopeptides detected displaying each high mannose glycan species.

Global, untargeted N-glycoproteomics analysis identified and quantified 1,966 individual N-glycopeptides. These peptides displayed 254 distinct N-glycan compositions and were distributed over 379 glycosylation sites across 305 unique glycoprotein species (Fig. 1B). Regarding the classes of glycans, high mannose glycans (immature glycans containing 6 to 9 terminal mannose residues) were present on ~27% of glycopeptides, while complex (mature glycans featuring terminal N-acetylglucosamine (GlcNAc), galactose, or sialic acid residues) and hybrid glycans (glycans terminating in both mannose and GlcNAc/galactose/sialic acid) were present in ~24% of glycopeptides. Additionally, many glycopeptides (31%) contained fucosylated glycans, whereas 13% exhibited sialylation, and only 5% lacked both fucosylation and sialylation (Fig. 1C). Interestingly, among the identified 1,429 complex/hybrid glycopeptides, 429 possessed bisected glycan structures, which is commonly found in the brain. Within the 527 glycopeptides bearing high mannose glycans, 155 contained a Man_5_ (5 mannose residues) composition, followed by 74 glycopeptides each possessing Man_6_ and Man_8_ glycans (Fig. 1D).

### MDD and BDD exhibit increased protein N-glycosylation.

**Figure 2:**
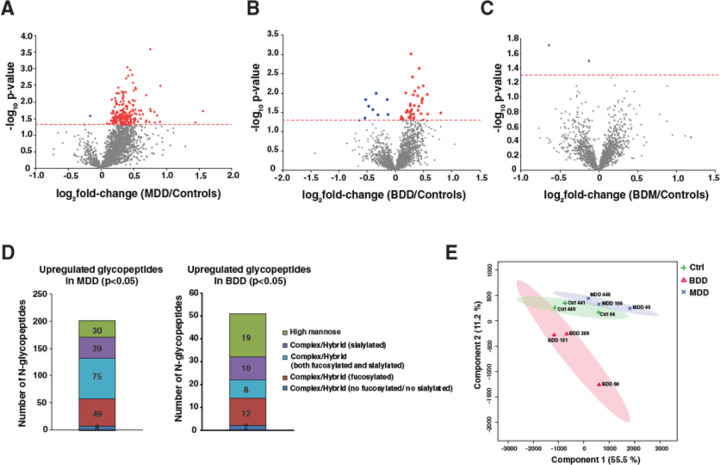
Glycoproteome alterations in brain samples in individuals with different mood disorders. **A.** Volcano plot depicting the differentially expressed glycopeptides in individuals with major depressive disorder (MDD). **B.** Volcano plot depicting the differentially expressed glycopeptides in individuals with bipolar depression (BDD). **C.** Volcano plot depicting the differentially expressed glycopeptides in individuals with bipolar mania (BDM). X-axis is log_2_ fold-change of individuals with MDD, BDD, or BDM versus controls, and Y-axis is the negative logarithm of p-value from a t test for significance as indicated. The horizontal dashed red line represents the cutoff for significance (p=0.05). **D.** Stacked bar chart displaying the number of significantly enriched glycopeptides displaying glycans of different classes in individuals with MDD and BDD. **E.** Partial Least Squares Discriminant Analysis (PLS-DA) based on reporter ion intensities for all identified glycopeptides in individuals with MDD, BDD, and controls. The percentage of total variance associated with each component is shown in brackets with the axis label.

Glycoproteomic analysis revealed increased protein N-glycosylation in DLPFC samples from both individuals with MDD and individuals with BD who were depressed at TOD. Remarkably, in the DLPFC of individuals with MDD, among the 1,966 identified glycopeptides, 199 unique N-glycopeptides derived from 69 unique glycoproteins displayed significant (p<0.05) enrichment relative to controls, while only two glycopeptides displayed significant depletion, consistent with a profound hyperglycosylation phenotype (Fig. 2A). In DLPFC from individuals with bipolar depression, among the 1,966 identified glycopeptides, only 42 N-glycopeptides derived from 28 distinct glycoproteins were significantly enriched relative to controls (Fig. 2B). Conversely, in DLPC of individuals BD who were manic at TOD, no significantly altered glycosylation was observed (Fig. 2C).

Among the enriched glycopeptides in individuals with unipolar depression, complex/hybrid glycopeptides, both fucosylated and sialylated, were the most abundant, while complex/hybrid glycopeptides with only fucosylation were the second most abundant. Of the enriched 42 glycopeptides in the DLPFC of individuals with bipolar depression, the most abundant enriched glycopeptides possessed high mannose glycans, while the second most abundant glycopeptides were fucosylated complex/hybrid glycopeptides (Fig. 2D). Partial least squares-discriminant analysis (PLS-DA) of the glycoproteome revealed a clear separation between unipolar and bipolar depression samples (Fig. 2E).

### Several critical brain proteins showed increased glycosylation in MDD and BDD

Increased N-glycosylation was observed in several critical brain glycoproteins. These altered glycopeptides exhibited varying glycan compositions ranging from paucimannose (less than 4 terminal mannose residues), high mannose (4 mannose residues and higher), N-acetylglucosamine (GlcNAc) bisected, di-sialylated (diSia), and sialylated and fucosylated complex/hybrid glycans.

**Figure 3:**
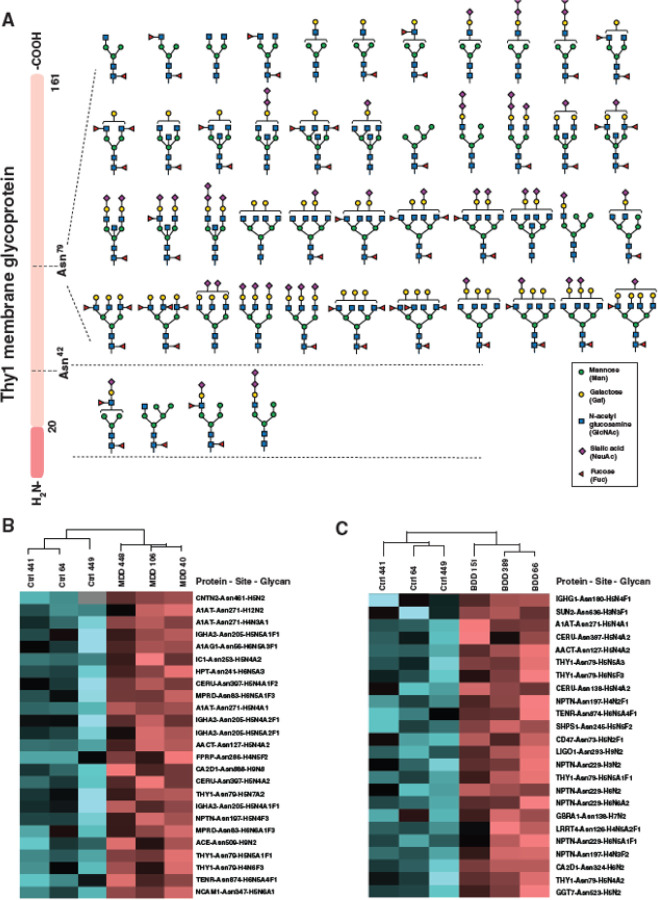
Significantly increased glycopeptides in Thy1 membrane glycoprotein and site-specific glycosylation changes in depression. **A.** Thy1 membrane glycoprotein (THY1) sequence is illustrated with two N-glycosylation sites (Asn^42^ and Asn^79^). The significantly enriched glycan structures in individuals with major depression (MDD) on these sites are illustrated, with most glycopeptides possessing the diSia and bisected glycan structures. Putative structures are shown using Symbol Nomenclature for Glycans (SNFG). **B,C.** Heatmaps of glycopeptide abundances which most discriminate individuals with MDD (**B**) and bipolar depression (BDD) (**C**) from controls. The pattern is color coded. Protein, glycosylation site, and glycan composition are indexed on the right of the heatmaps. Asn^x^ represents the asparagine at amino acid site “x” in the corresponding protein sequence. H=Hexose; N=N-acetylhexosamine; A=N-acetylneuraminic acid; F=Fucose.

The glycopeptides that exhibited the greatest enrichment in individuals with MDD were derived from the Thy-1 Cell Surface Antigen (THY1), where 48 glycopeptides were significantly enriched over two glycosylation sites, N79 and N42. Forty-four distinct glycan compositions were detected on N79: 4 glycopeptides diSia glycans with or without fucosylation, 4 diSia with GlcNAc bisected glycans, 7 GlcNAc bisected glycans, and 1 fucosylated high mannose (Man_5_) glycan. The most highly enriched glycopeptide on N79 displayed a complex sialylated glycan with a fold-change (FC) of 1.5, while and the second most highly enriched glycopeptide (FC 1.45) displayed a fucosylated and sialylated bisected glycan. The remaining four glycans were detected on N42, of which one glycan exhibited diSia glycan structure. The structures of these glycopeptides on both glycosylation sites of THY1 protein are illustrated in Fig. 3A.

Twelve significantly enriched glycopeptides derived from neuroplastin (NPTN), the cell recognition glycoprotein, across two glycosylation sites – N227 and N197 – were also observed in individuals with MDD. Of these, 11 glycans were enriched on site N229, while one glycan was enriched on N197. The most highly enriched NPTN glycopeptides displayed fucosylated bisected glycans (Hex_6_HexNAc_6_Fuc_2_) (F.C. 1.4), fucosylated triantennary glycans (Hex_6_HexNAc_5_Fuc_3_) (F.C. ~1.3), and fucosylated and sialylated triantennary glycans (Hex_6_HexNAc_5_NeuAc_2_Fuc_2_) (F.C. 1.3).

We also observed 11 enriched glycopeptides derived from the tenascin-R (TENR) extracellular matrix protein across three glycosylation sites (N55, N581,and N874). All TENR glycopeptides were either fucosylated or sialylated or both. Additional enriched glycopeptides observed were derived from cell adhesion molecule 3 (CADM3; 7 glycopeptides with 7 glycan compositions across 1 site), voltage-dependent calcium channel subunit alpha-2/delta-1 (CA2D1; 6 glycopeptides with 6 glycan compositions across 3 sites), cell adhesion molecule 1 (CADM1; 5 glycopeptides with 5 glycan compositions across 3 sites) and neural cell adhesion molecule 1 (NCAM1; 5 glycopeptides with 5 glycan compositions across 2 sites). The expression patterns of the most significantly enriched glycopeptides from both individuals with MDD and controls are depicted in a heatmap showing their clustering within the groups (Fig. 3B).

In individuals with bipolar depression, the glycopeptides displaying the greatest enrichment were derived from NPTN, with 7 glycopeptides enriched across two glycosylation sites, N197 and N229. At N197 the core fucosylated Man_4_ and a fucosylated biantennary glycans (Hex_4_HexNAc_3_Fuc_2_) were enriched FC ~1.4 and ~1.25 respectively. At N229 5 glycopeptides were enriched displaying high mannose glycans (Man_5_, Man_6_), a paucimannose glycan (Man_3_), a sialylated bisected glycan (Hex_6_HexNAc_6_NeuAc_2_) and a fucosylated and sialylated triantennary glycan (Hex_6_HexNAc_5_NeuAc_1_Fuc_1_). The THY1 glycoprotein also displayed increased N-glycosylation in individuals with bipolar depression, with 4 glycopeptides enriched at N79. These glycopeptides displayed one sialylated biantennary glycan (Hex_5_HexNAc_4_NeuAc_2_) (F.C. 1.2), one diSia bisected glycan (Hex_5_HexNAc_5_NeuAc_3_) (F.C. 1.4), one triantennary fucosylated glycan (Hex_6_HexNAc_6_Fuc_3_) (F.C. 1.4) and one fucosylated, sialylated bisected glycan (Hex_5_HexNAc_5_NeuAc_1_Fuc_1_) (F.C. 1.3).

Additional glycopeptides enriched in individuals with bipolar depression were derived from CA2D1 (3 glycopeptides with 3 glycan compositions across 1 site), tyrosine-protein phosphatase non-receptor type substrate 1 (SHPS1; 3 glycopeptides with 3 glycan compositions across 2 sites), ceruloplasmin (CERU; 2 glycopeptides with 2 glycan compositions across 2 sites), leukocyte surface antigen CD47 (2 glycopeptides with 2 glycan compositions across 1 site) and brevican core protein (PGCB; 2 glycopeptides with 2 glycan compositions across 1 site). The enrichment patterns of glycopeptides in bipolar depression and control groups are depicted in Fig. 3C.

**Figure 4:**
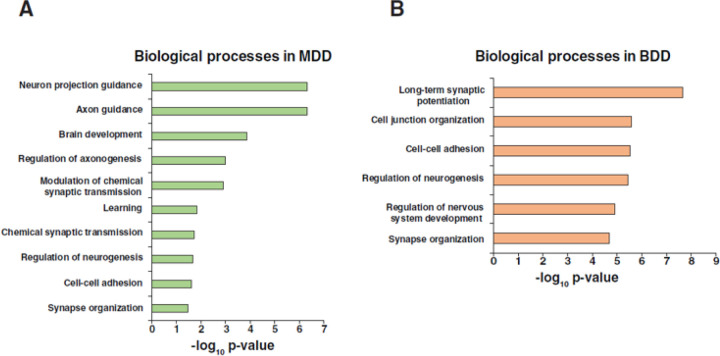
Gene ontology analysis of biological processes involving differentially enriched glycoproteins. **A.** Top enriched biological processes in individuals with major depression (MDD). **B.** Top enriched biological processes in individuals with bipolar depression (BDD). X axis is the negative logarithm of p-values for significance.

Gene Ontology (GO) analysis of the differentially N-glycosylated glycoproteins demonstrated that neuronal projection guidance was among the most enriched biological processes in the DLPFC of individuals with MDD (Fig. 4A). Other highly enriched processes included axon guidance, brain development, axonogenesis, neurogenesis, modulation of chemical synaptic transmission, cell-cell adhesion and synaptic organization. The most enriched biological processes in the DLPFC of individuals with bipolar depression included long term synaptic potentiation and cell junction organization (Fig. 4B). There was substantial overlap in top-ranked GO biological processes between unipolar and bipolar depression, including cell-cell adhesion, regulation of neurogenesis, and synaptic organization. A comprehensive list of all the identified glycopeptides with glycosylation sites and glycan compositions interrogated with the fold-changes and p-values can be found in Supplementary Table 1.

### Comparative glycoproteome between different mood disorders

**Figure 5:**
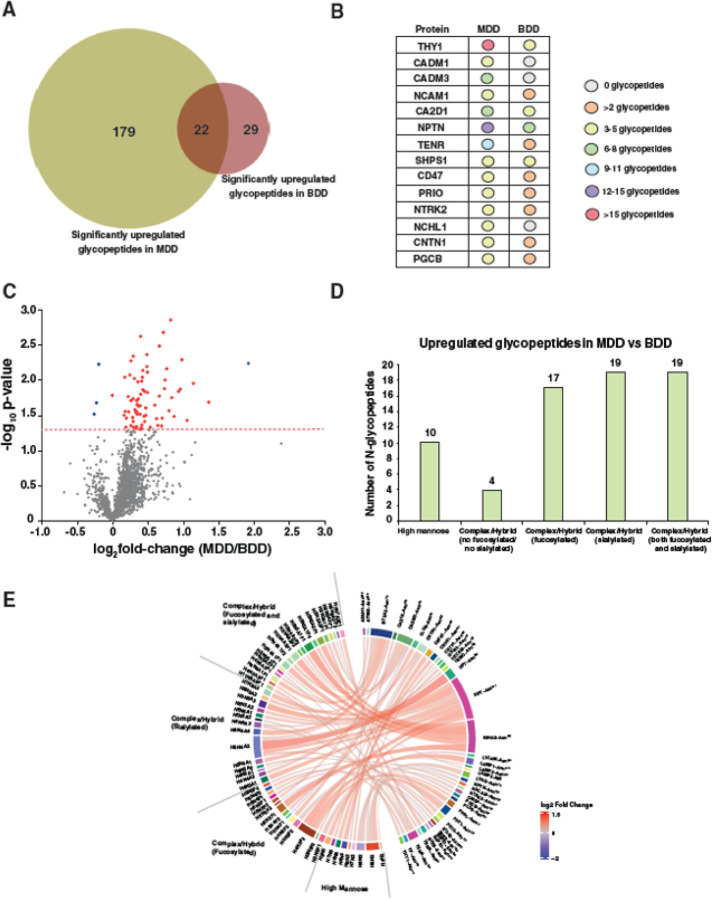
Comparative glycoproteome between major depression disorder and bipolar depression depressive. **A.** Venn diagram of significantly enriched (p<0.05) N-glycopeptides between individuals with major depression (MDD) and bipolar depression (BDD) demonstrating the numbers of common and uniquely enriched N-glycopeptides. **B.** Numbers of significantly enriched glycopeptides (p<0.05) corresponding to the proteins in individuals with MDD and BDD are represented with different colors corresponding to glycopeptide enrichment. **C.** Volcano plot depicting enriched and depleted glycopeptides in individuals with MDD relative to BDD. X-axis is log_2_ fold-change individuals with MDD relative to controls and Y-axis is the negative logarithm of p-value from a t test for significance as indicated. The horizontal dashed red line represents the cutoff for significance (p=0.05). **D.** Bar graph of the number of glycopeptides significantly enriched (p<0.05) in individuals with MDD relative to BDD organized by glycan structure. **E.** Differential chord diagram depicting the site-specific glycosylation changes for proteins encompassing significantly changing glycopeptides in individuals with MDD relative to BDD. Proteins with different glycosylation sites are indexed on the right of the diagram and connected via chords to respective identified glycan structures on the left. Asn^x^ represents the asparagine at amino acid site “x” in the corresponding protein sequence. H=Hexose; N=N-acetylhexosamine; A=N-acetylneuraminic acid; F=Fucose.

When we compared the significantly enriched glycopeptides between individuals with unipolar and bipolar depression, only 22 glycopeptides were found to be commonly enriched in both groups. 179 unique glycopeptides were enriched solely in MDD, while only 29 glycopeptides were solely enriched in bipolar depression (Fig. 5A). Many shared upregulated glycopeptides in unipolar and bipolar depression were derived from multiple glycosylation sites of NPTN, THY1, CA2D1, and SHPS1 proteins. Although less abundant, additional shared upregulated glycopeptides derived proteins included NCAM1, TENR, CD47, contactin-1 (CNTN1), and PGCB (Fig. 5B). A comprehensive list of shared enriched glycopeptides in unipolar and bipolar depression with glycosylation sites and glycan compositions interrogated with the fold-change and p-values can be found in Supplementary Table 2.

Next, we compared the abundances of glycopeptides between individuals with unipolar and bipolar depression. Increased N-glycosylation was observed in all individuals with MDD relative to individuals with bipolar depression, across numerous glycosylation sites on multiple glycoproteins (Fig. 5C). Among the 1,966 identified glycopeptides, significant glycosylation changes (p < 0.05) were observed in 73 unique N-glycopeptides derived from 35 distinct glycoproteins. The highest numbers of enriched glycopeptides displayed complex/hybrid glycans with either sialylation and/or fucosylation. Ten glycopeptides displayed high mannose glycans and only 4 glycopeptides displayed complex/hybrid glycans without fucosylation or sialylation (Fig. 5D). 70 glycopeptides were enriched in individuals with MDD, the most significant of which (F.C. ~2.5) was derived from haptoglobin (HPT) and displayed a biantennary glycan (Hex_5_HexNAc_4_NeuAc_2_). The next three top enriched glycopeptides were all derived from immunoglobulin heavy constant alpha 2 (IGHA2), and displayed a sialylated bisected glycan (Hex_5_HexNAc_5_NeuAc_1_Fuc_1_), a fucosylated bisected glycan (Hex_5_HexNAc_5_NeuAc_1_Fuc_1_) and a biantennary glycan (Hex_5_HexNAc_4_NeuAc_2_Fuc_1_). A differential chord diagram is drawn to quantitatively visualize glycosylation differences in MDD relative to bipolar depression with different glycan compositions on distinct glycosylation sites of various cellular proteins (Fig. 5E).

DLPFC of individuals with bipolar depression also displayed increased N-glycosylation relative to those from individuals with BD who were manic at TOD. Out of the 1,966 glycopeptides identified, only 9 showed significant enrichment or depletions. These glycopeptides included once enriched glycopeptide from NPTN (F.C. 1.3) displaying a high mannose glycan, and on enriched glycopeptide from THY1 (F.C. 1.3) displaying a bisected sialylated glycan. Several additional glycopeptides were also increased in individuals with BD in a depressed mood state relative to those in a manic state, however, these did not reach statistical significance. A comprehensive list of all identified glycopeptides with glycosylation sites and glycan compositions interrogated with the fold-changes and p-values can be found in Supplementary Table 1.

## Discussion

Multiomics approaches have previously been leveraged to explore the etiology of MDD and BD. Combining genome-wide association study (GWAS) data with metabolomics has revealed distinct biosignatures for early-onset and adult-onset MDD, highlighting potential new markers for MDD susceptibility and resilience (Grant, Barreto et al. 2022). Another integrative and comparative proteomic and metabolomic study, coupled with network analysis, demonstrated connections between proteins and metabolites, suggesting possible alterations in the hemostasis of BD patients (Ribeiro, Sen et al. 2022). Additionally, proteomic analysis from 270 individuals with MDD and BD, along with healthy controls, identified nine proteins associated with neuro, oxidative/nitrosative stress, and immunity/inflammation-related biological functions. This analysis provided proteomic signatures of potential differences between MDD and BD (Shin, Rhee et al. 2021).

Our combined analysis, including a literature review and natural history data, provides a detailed understanding of depression in CDG. The CDC reports a 4.4% prevalence of depression in children aged 3–17 years in the US from 2016–2019 (https://www.cdc.gov/childrensmentalhealth/features/anxiety-depression-children.html). The age-standardized prevalence of depression among adults was 18.5% in 2020 (https://www.cdc.gov/mmwr/volumes/72/wr/mm7224a1.htm). In contrast, the frequency of depression in the CDG literature cohort in children living with a chronic disease, was much lower (0.07%), and in our natural history study, it was 1.8%. However, we note that standardized diagnostic criteria for assessing comorbid depression in CDG are not part of routine clinical practice, which may have led to an underestimation of depression frequency in both the literature and patient records.

CDG is a chronic, debilitating multisystem disorder with significant central nervous system involvement (Lam, Scaglia et al. 2024). Previous research on chronically ill children suggests they are at a slightly higher risk for depressive symptoms (Morava, Gardeitchik et al. 2010). However, the frequency of major depressive disorder (MDD) in various chronic disorders ranges from 3–26%, with a 2–5 times increased risk in those with severe central nervous system conditions such as epilepsy, psychosis, and mitochondrial disease (Wray and Radley-Smith 2004, Morava, Gardeitchik et al. 2010).

Our comprehensive analysis of the global N-glycoproteome in postmortem brain samples revealed a predominance of Man_5_ structures and fucosylated/bisected complex/hybrid structures. These findings align with previous findings on the brain glycoproteome (Lee, Ha et al. 2020, Williams, Noel et al. 2022). Furthermore, our investigation identified an increase in various glycan structures across important brain proteins in individuals with both unipolar and bipolar depression. No evidence was found to suggest a reduction in N-glycosylation across any glycoforms of these proteins. The proteomic analysis conducted on these samples demonstrated that there were no changes in the expression of these proteins (data not shown), implying that the observed rise in glycosylation is not a result of changes in protein levels. These proteins play crucial roles in brain function and neuronal development. Notably, we observed a more substantial increase in global glycosylation in unipolar depression relative to bipolar depression, while no significant glycosylation differences were observed in individuals with mania. The increased N-glycosylation in bipolar depression relative to mania suggests that a complete rewiring of protein N-glycosylation is necessary from transitioning between the depressed and manic mood states. It also suggests that there might be glycosylation normalization in depressed individuals when transitioning back to a manic state.

Among the identified glycoproteins, Thy1 membrane glycoprotein (THY1) exhibited the highest level of hyperglycosylation in both unipolar and bipolar depression. THY1 is a small glycosylphosphatidylinositol (GPI)-anchored protein located in the outer membrane, participating in cell adhesion and cell communication (Herrera-Molina, Valdivia et al. 2013, Ilic, Auer et al. 2019). We also identified the presence of diSia-containing glycans on THY1, which displayed a significant increase in individuals with unipolar depression but only a subtle change in individuals with bipolar depression. These diSia structures play a vital role in central nervous system maturation, contributing to neuronal plasticity and synaptic connectivity (Schnaar, Gerardy-Schahn et al. 2014, Hildebrandt and Dityatev 2015). Polysialylated neuronal cell adhesion molecules are prominently involved in the developing nervous system and have been extensively studied (Seki and Arai 1993, Hildebrandt, Muhlenhoff et al. 2007). Additionally, we observed an elevation in different GlcNAc bisected glycans on THY1, a glycan modification predominantly found in the brain.

Another noteworthy finding was the dysregulated glycosylation of neuroplastin, which was significantly increased on multiple sites in both unipolar and bipolar depression. Neuroplastin belongs to the cell-adhesion molecule family located on synaptic membranes and is associated with memory formation and cognition (Ilic, Mlinac-Jerkovic et al. 2021). Hyperglycosylation of another extracellular matrix protein tenascin-R, was observed in both unipolar and bipolar depression. Tenascin-R is exclusive to the nervous system, which is known to regulate axon extension and organization, myelinization, and perineuronal net formation, and its expression has been associated with several neurodegenerative disorders (Anlar and Gunel-Ozcan 2012). We hypothesize that the increased glycosylation of these proteins may have altered their activities, which may have an impact on the pathophysiology of mood disorders.

One of the top significantly enriched glycans in individuals with BDD was at N138 of GBRA1. GBRA1 is the α1 subunit of the ionotropic γ-aminobutyric acid (GABA) receptor. GABA is the primary inhibitory neurotransmitter in the mammalian brain, and unsurprisingly has been implicated in both MDD {Luscher, 2011 #87} and BD. The ionotropic GABA receptors form a ligand gated ion channel upon agonism. The activity of ionotropic GABA receptor subunits, including α1 has been shown to be modulated by N-glycosylation, which has been shown to be altered in schizophrenia, with some subunits being shown to be hyperglycosylated, while others are shown to be hypoglycosylated (Mueller, Haroutunian et al. 2014). These data implicate GABA receptor subunit hyperglycosylation in BD, particularly bipolar depression.

In a comparative mass spectrometry-based N-glycoproteomic analysis of post-mortem brain samples of individuals with Alzheimer’s disease (AD) revealed alterations in N-glycoprotein abundance and site occupancy affecting various processes and pathways including cell adhesion, synaptic dysfunction, cell signaling, and neuroinflammation (Zhang, Ma et al. 2020). A similar analysis in symptomatic and asymptomatic AD found that the Man_5_ N-glycan and low sialylation were frequently observed in the brain tissues along with AD-associated glycoproteomic changes that could provide better understanding in disease progression (Suttapitugsakul, Stavenhagen et al. 2022). An increased N-glycosylation of several glycans including bisected N-acetylglucosamine (GlcNAc) have been observed in cerebrospinal fluid and brain samples from patients with AD (Schedin-Weiss, Gaunitz et al. 2020). Similarly, in our data, the increased abundance of bisected GlcNAc glycans was found in individuals with both unipolar and bipolar depression, suggesting that protein glycosylation plays a common role for the progression of depressive symptoms in both disorders.

In a previous study, depletion of polysialylated NCAM1 (PSA-NCAM) was observed in dorsolateral prefrontal cortex of post-mortem samples in individuals with schizophrenia, and this change was not observed in cases of BD or MDD (Gilabert-Juan, Varea et al. 2012). N-glycomic study of CSF samples demonstrated the reduction of bisected and sialylated N-glycans but increase in specific complex N-glycans in individuals with schizophrenia (Stanta, Saldova et al. 2010). The abnormal glycosylation has been proposed to be a fundamental factor affecting several processes such as oxidative stress and inflammation, regulating mitochondrial dynamics in brain tissue in Parkinson’s disease (Videira and Castro-Caldas 2018). Furthermore, Sandi et al. (Sandi, Merino et al. 2001) reported that NCAM1 and its polysialylated form may play a role in hippocampal structural remodeling following chronic stress, a key vulnerability for developing depression.

Both AD and Parkinson’s disease represented dysregulated glycosylation in proteins associated with extracellular matrix dysfunction, cell adhesion, neuroinflammation, synaptic dysfunction, and cell signaling. Our findings also showed the altered N-glycosylation in these processes suggesting potential shared underlying pathomechanism in various neuropsychiatric disorders. Although the glycosylation of these biological processes was commonly dysregulated in both unipolar and bipolar depression, protein glycosylation of other processes such as learning, neuron projection guidance and axon guidance were only altered in MDD.

A recent study by Yang et al. used RNA sequencing of human post-mortem brains and mice exposed to chronic stress, finding a positive correlation between protein N-glycosylation and depression (Yang, Li et al. 2024). They also showed that inhibiting N-glycosylation improved depressive-like behavior. Boeck et al. further reported that abnormal N-glycosylation was linked to the severity of MDD symptoms in females (Boeck, Pfister et al. 2018). Collectively, these findings highlight the critical role of brain protein N-glycosylation in depression pathology.

## Conclusion and Significance of the Findings

Our study provides a comprehensive analysis of depression rates among individuals with CDG, showing significantly lower levels of depression in this group compared to the general population and those with other chronic illnesses. This lower prevalence of depression in the CDG cohort, despite the involvement of the central nervous system, suggests that glycosylation defects may offer a protective effect against developing depression. Furthermore, our glycoproteomic analysis reveals notable alterations in N-glycosylation patterns across different mood disorders. The increased N-glycosylation found particularly in MDD and BDD indicates that N-glycosylation is a key factor in the pathophysiology of these disorders. We suggest that inhibiting N-glycosylation could be a promising new approach for therapeutic interventions targeting disease-specific glycosylation in depressive mood disorders.

## Materials and Methods

### Systematic literature search

PubMed and Embase searches were conducted on 08-03-2024 using the keywords “depress*,” “CDG,” and “congenital disorder of glycosylation,” with no date restrictions. Two independent researchers reviewed and scored articles based on inclusion and exclusion criteria. Duplicates were removed, and relevant articles addressing both congenital disorders of glycosylation and psychopathology were selected for inclusion.

### Exclusion Criteria:

- No official diagnosis of psychopathology.
- CDG or related terminology not mentioned in the title or abstract.
- Non-human studies.
- Articles in foreign languages.

### Assessment of depression in the FCDGC natural history study

We reviewed natural history data of 110 CDG patients with confirmed pathogenic variants, enrolled in the Frontiers of Congenital Disorders of Glycosylation Consortium (FCDGC) natural history study (IRB: 19–005187; NCT04199000) at Mayo Clinic and Mount Sinai Hospital over the past year. We evaluated all CDG types enrolled in the CDG natural history study at these two sites. The diagnosis of depression was established based on natural history patient records and confirmed by a clinician.

### Natural history Study

The FCDGC Natural History (NH) study is an NIH-funded collaborative effort. To enroll, participants visit with one of the site study teams either in person or virtually for a review of medical history, physical examination, and laboratory tests. They and/or their legally authorized representative provided written or electronically signed consent and/or assent using an IRB-approved consent/assent tool as indicated. All procedures were in accordance with the ethical standards of the Helsinki Declaration of 1975, as revised in 2013. Clinical data from clinical interview, previous history including notes, physical examination, and caregiver proxy PROMIS questionnaires [http://www.hqlo.com/content/10/1/22], were collected and recorded into the REDcap database hosted on the NIH Cloud managed by the Data Management and Coordinating Center at the Children’s Hospital Medical Center and the University of Cincinnati.

### Post-mortem brain sample collection and cohort information

Post-mortem dorsolateral prefrontal cortex (DLPFC) biopsies from 3 individuals with MDD, 6 individuals with BD, and 3 controls were acquired. 3 of the individuals with BD were determined to be in a depressed mood state at time of death (TOD), while the remaining 3 were determined to be manic at TOD. All DLPFC samples were collected from the Douglas Bell Canada Brain Bank and University Hospitals Cleveland Medical Center. All procedures performed were in accordance with the Declaration of Helsinki and had been approved by the Institutional Review Boards of the University Hospitals Cleveland Medical Center, Cleveland, OH (11-88-233). Psychological autopsies are conducted with best informants to obtain histories of psychopathology and other relevant information as described elsewhere (Dumais, Lesage et al. 2005, Ho, Winham et al. 2019). All brain samples with different mood disorders include the age, cause of death (COD), pH and medication clinical features are provided in Table 1.

### Protein isolation and digestion

Brain samples from different mood disorders were lysed in 5% SDS (in 100 mM TEABC)using a Bioruptor sonication device (Hologic). Protein concentrations in the obtained lysates were estimated using the Pierce BCA Protein Assay Kit per the manufacturer’s instructions (Thermo Fisher Scientific). Equal masses of protein were first reduced using 20 mM dithiothreitol (DTT) for 10 minutes at 95°C and then alkylated with 100 mM iodoacetamide (IAA) for 30 minutes in the dark at room temperature. Samples were subsequently digested with trypsin (Worthington) using S-Trap midi cartridges (ProtiFi) per the manufacturer’s instructions. The peptide concentration was again quantified and equal amounts of peptide were labeled with tandem mass tags (TMT) (Thermo Fisher Scientific) for a multiplex mass spectrometry experiment as per the manufacturer’s protocol.

### Glycopeptide enrichment

The TMT-labeled and pooled peptides were resuspended in 0.1% formic acid and injected into Superdex peptide 10/300 column (GE Healthcare) as described previously (Saraswat, Garapati et al. 2021). The peptides were separated using an isocratic flow of 0.1% formic acid for 130 minutes and 22 early fractions were collected starting at 10 minutes after injection. The fractions were lyophilized and resuspended in 0.1% formic acid for LC-MS/MS analysis.

### LC-MS/MS based N-glycoproteomics analysis

LC-MS/MS analysis of fractionated samples obtained from size exclusion chromatography was carried out as previously described with some modifications (Ligezka, Radenkovic et al. 2021, Balakrishnan, Altassan et al. 2023, Budhraja, Saraswat et al. 2023, Budhraja, Joshi et al. 2024). Briefly, 12 concatenated fractions from SEC were analyzed on an Orbitrap Eclipse mass spectrometer (Thermo Fisher Scientific). Peptides were separated by liquid chromatography on an EASY-Spray column (75 μm × 50 cm, PepMap RSCL C18, Thermo Fisher Scientific). Peptides were first trapped on a trap column (100 mm × 2 cm, Acclaim PepMap100 Nano-Trap, Thermo Fisher Scientific) at a flow rate of 10 μL/min. LC separation was performed at a flow rate of 300 nL/min for 150 min using a linear gradient of 0.1% formic acid in water (solvent A) and 0.1% formic acid in acetonitrile (solvent B). All experiments were done in DDA mode at an isolation window of 0.7 m/z. Precursor ions were acquired at a resolution of 120,000 (at m/z 200) and fragment ions at a resolution of 30,000 (at m/z 200). The fragmentation was carried out using stepped higher-energy collisional dissociation (HCD) method (15, 25, 40%). A schematic of the optimized workflow for multiplexed glycoproteomics is shown in Fig. 1A.

### Data analysis

Data analysis was performed as described previously (Ligezka, Radenkovic et al. 2021, Balakrishnan, Altassan et al. 2023, Budhraja, Saraswat et al. 2023). The data was searched using the publicly available software pGlyco version 3.0 (Zeng, Cao et al. 2021) with an in-built human N-glycan database for identifying glycans and Uniprot Human Reviewed protein database for identifying peptide sequence. False discovery rate (FDR) was set to 1% at the peptide-spectrum matches (PSMs), peptide, protein, and glycopeptides levels. To quantify glycopeptides, reporter ion quantification was performed for glycoproteomics raw files in Proteome Discoverer v2.5 and glycopeptide IDs obtained from pGlyco3 were matched with quantitation on a scan-to-scan basis. All data are expressed as mean±SD. Statistical analysis was performed using the publicly available computational platform Perseus (Tyanova, Temu et al. 2016), MetaboAnalyst (Pang, Zhou et al. 2022), and R studio. Two sample t tests were used to compare two groups. Gene Ontology analysis of proteins corresponded to differentially expressed glycopeptides to identify functionally relevant biological processes dysregulated in MDD and BDD individuals.

## Figures and Tables

**Table 1.** Mood disorder patients and their clinical features.

Sample ID	Gender	Age	COD	pH	Simplified Axis 1	Substance at Death	Last 3 months medications
MD40	Male	49	Suicide	6.57	Major depressive disorder	Nil	N/A
MD106	Male	39	Suicide	6.37	Major depressive disorder	Nil	Antidepressants (SNRI), Antipsychotics
MD448	Male	48	Suicide		Major depressive disorder	Nil	Nil
BD66	Male	28	Suicide	6.51	Bipolar I disorder (depression)	Nil	Antidepressants (SSRI), Antipsychoticss
BD389	Female	42	Suicide	6.2	Bipolar disorder (depression)	Antidepressant (NaSSA)	Antiepileptic, Antidepressant (TCA), Benzodiazepine, Mood stabilizer
BD151	Female	27	Accidental	5.98	Bipolar I disorder (depression)	N/A	Antipsychotics, Antimanic
BDM121	Male	32	Suicide	6.75	Bipolar I disorder (mania)	Cocaine and metabolites, Alcohol, Antidepressants (SNRI)	Antidepressants (SNRI), Antipsychotics, Benzodiazepines
BDM144	Female	38	Suicide	6.92	Bipolar I disorder (mania)	Antipsychotics	Antipsychotics
BDM442	Female	47	Accidental	6.34	Bipolar Disorder (mania)	Cocaine metabolites, Methamphetamin, Antipsychotic	Antipsychotics, Injections
HE64	Male	64	Natural	5.65	Nil	Nil	N/A
HE441	Male	36	Accidental	6.32	Nil	Nil	Nil
HE449	Male	54	Accidental	6.3	Nil	Nil	Nil

**Table 2.** Depression in specific CDG types. The numbers represent patients with depression identified in the literature search. Percentages reflect the estimated frequency of depression based on the total number of case reports. The bottom row shows the total number of patients with depression and the overall frequency of depression across all patients.

Gene	Co-morbid depression	Total No. of Case reports	References
*PMM2*	1 / 0.1%	900	(Monin, Mignot et al. 2014, Altassan, Péanne et al. 2019)
*COG6*		16	(Xia, Ng et al. 2023)
*PIGV*		32	(Hutny, Lipinski et al. 2023)
*MAN1B1*		42	(Kasapkara, Olgac et al. 2021)
*STT3A*		16	(Wilson, Garanto et al. 2021)
*ALG13*		83	(Shah, Johnsen et al. 2023)
*ALG12*		12	(Shanmugasundaram, Wang et al. 2024)
*ALG11*		12	(Erdal, Ceylan et al. 2023)
*ALG6*		41	(Morava, Tiemes et al. 2016)
*ALG8*		26	(Uzunyayla-Inci, Kiykim et al. 2023)
*ALG1*		43	(Öncül, Kose et al. 2022)
*SRD5A3*		38	(Jaeken, Lefeber et al. 2020)
*PGAP3*		65	(Altassan, Allers et al. 2023)
*MAGT1*		22	(Watson, Nadat et al. 2022)
*DOLK*		20	(Rush, Baker et al. 2017)
*DPAGT1*		26	(Özsoy, Cinleti et al. 2023)
*RFT1*		16	(Quelhas, Jaeken et al. 2019)
*SLC35C1*		19	(Hüllen, Falkenstein et al. 2021)
*SLC35A3*		2+1+8+1+1 = 13	(Edvardson, Ashikov et al. 2013, Edmondson, Bedoukian et al. 2017, Marini, Hardies et al. 2017, Miyake, de Oliveira Stephan et al. 2021, Quelhas, Correia et al. 2021)
*LADII*		66	(Quelhas, Correia et al. 2021)
*SSR4*		22	(Johnsen, Tabatadze et al. 2024)
**Total / Average %**	**1 / 0.07%**	**1530**	

**Table 3.** Comorbid depression in individuals with CDG. The numbers represent patients with reported comorbid depression. The last row shows the frequency of depression relative to the entire cohort of CDG patients (N=110).

Gene	Co-morbid depression	Total # of individuals in Natural History Study of FCDGC
*PMM2*	0	55
*MPI*	0	1
*ALG12*	1	1
*ALG13*	0	1
*ALG1*	0	2
*ALG6*	0	1
*ALG8*	0	1
*ATP6AP1*	0	1
*SLC35C1*	0	2
*DDOST*	0	1
*DPM1*	0	1
*GMPPA*	0	1
*GPAA1*	0	1
*OGT*	1	4
*PGT*	0	3
*PGO*	0	1
*PGN*	0	2
*PIGS*	0	1
*PGW*	0	1
*PGA*	0	2
*PGV*	0	1
*PGAP3*	0	1
*PGM1*	0	5
*SSR4*	0	3
*SLC39A8*	0	2
*SLC35A2*	0	2
*SLCWA7*	0	1
*DHDDS*	0	4
*MAN1B1*	0	2
*NGLY1*	0	1
**Total**	**2/110 (1.8%)**	**110**
